# Macular hyperpigmentary changes in *ABCA4*-Stargardt disease

**DOI:** 10.1186/s40942-019-0160-4

**Published:** 2019-04-01

**Authors:** Maria Fernanda Abalem, Amro A. Omari, Dana Schlegel, Naheed W. Khan, Thiran Jayasundera

**Affiliations:** 1 Department of Ophthalmology and Visual Sciences, W. K. Kellogg Eye Center, University of Michigan, 1000 Wall Street, Ann Arbor, MI 48150 USA; 2 Department of Ophthalmology and Otolaryngology, University of Sao Paulo Medical School, Sao Paulo, São Paulo, Brazil

**Keywords:** Stargardt disease, Age related macular degeneration, Prognosis, Optical coherence tomography, Genetics

## Abstract

**Background:**

Stargardt disease (STGD) and age-related macular degeneration (AMD) share clinical and pathophysiological features. In AMD, macular hyperpigmentary changes are associated to a worse prognosis. The purpose of this study was to characterize macular hyperpigmentary changes in patients with STGD and associate them with the severity of phenotype.

**Materials and methods:**

This retrospective cross-sectional study included 141 patients with STGD. Hyperpigmentary changes were evaluated on color fundus photography and spectral-domain optical coherence tomography. Severity of phenotype was assessed by full-field electroretinogram (ffERG) and fundus autofluorescence (FAF) patterns, and visual acuity (VA).

**Results:**

Thirty patients (21.7%) showed macular hyperpigmentary changes in four distinct patterns. Out of seventeen patients who had follow-up images, eleven patients demonstrated increases of the hyperpigmented lesions, and progression of the underlying RPE atrophy overtime. VA remained stable. Of 28 patients who had ffERG, 17 patients presented with reduction of photopic and scotopic responses, while 8 presented with reduction of photopic responses only, and 3 presented with preserved photopic and scotopic responses. Of 25 patients who had FAF available, 12 presented with widespread disease extending anteriorly to the vascular arcades, while eight presented with widespread disease, extending beyond the vascular arcades, and 5 presented with disease confined to the foveal area.

**Conclusion:**

In this study, we demonstrated that patients with STGD with macular hyperpigmented lesions had a severe phenotype. Overtime, hyperpigmented lesions increased in size, spread across the retina, and migrated to different retinal layers. Macular hyperpigmentation may be a marker of advanced stage of the disease.

## Introduction

Macular atrophy is a manifestation of Stargardt disease (STGD) and of age-related macular degeneration (AMD) [1]. STGD is the most common inherited macular dystrophy in both children and adults, caused by pathogenic variants in the *ABCA4* gene [2]; whereas AMD is a multifactorial disease and the leading cause of blindness in the elderly [3]. Many factors are known to be associated with the physiopathology underlying the development of retinal pigment epithelium (RPE) atrophy in patients with STGD and AMD, and there is evidence that the toxic accumulation of lipofuscin and its bis-retinoid components, including *N*-retinylidene-*N*-retinylethanolamine (A2E), play a role in both conditions [4–6]. The A2E accumulates in the RPE and photoreceptors, leading to the degeneration of these cells [4, 5, 7].

Clinically, STGD and AMD also share common features, such as decreased central vision, bilaterality, and macular atrophy. In STGD, the macular atrophy is usually symmetric and may be accompanied by flecks [8, 9]. Two prognostic factors have been associated with disease progression: fundus autofluorescence (FAF) and full-field electroretinogram (ffERG) patterns at baseline [10, 11]. Patients with multiple and widespread lesions on FAF and with cone-rod dysfunction on ffERG tend to exhibit a more severe phenotype than do those with localized macular changes and normal function of rods and cones [10, 11]. In AMD, on the other hand, patients most commonly present with drusen and pigmentary changes and develop macular atrophy (dry AMD) and/or choroidal neovascularization (wet AMD) over time [12]. In AMD, three major factors have been associated to the disease progression: the presence of large drusen [13], large drusen areas, and pigmentary changes [12, 14–17].

In AMD, pigmentary changes either consist of hypopigmentation represented by depigmented areas not related to geographic atrophy or by hyperpigmentation represented by deposits of gray-black pigment on the fundus exam [12, 18]. The hyperpigmentation is thought to result from RPE degeneration, displacement, or migration [19–21]. Clinical studies have corroborated this theory by demonstrating a correlation between pigment clumping observed on color fundus photography (CFP) with hyperreflective foci in several retinal layers, including the outer nuclear layer (ONL), outer plexiform layer (OPL), and inner nuclear layer (INL) on spectral domain optical coherence tomography (SD-OCT) [22, 23]. Although the association of hyperpigmentation with disease etiology in AMD has already been described, to our knowledge, the presence and significance of hyperpigmented changes and associated RPE changes in patients with STGD are still poorly defined. The aim of this study was to better characterize macular hyperpigmentary changes in patients with STGD and investigate the association of this finding with the severity of the clinical phenotype. We also provide some preliminary follow up data, which includes a comparison of the visual acuity at baseline and follow up, and the progression of both the number and density of macular hyperpigmentary changes on color fundus photography and migration from outer to inner retinal layers on SD-OCT.

## Materials and methods

A retrospective chart review was performed at the Kellogg Eye Center, at Michigan Medicine. The study was approved by the University of Michigan Institutional Review Board and conducted accordingly to the Declaration of Helsinki.

### Patient selection

We only included patients with both a clinical and genetic diagnosis of STGD. The clinical diagnosis was established by the presence of central vision dysfunction and bilateral macular atrophy, with or without surrounding flecks on fundus exam. Genetic diagnosis was established by the presence of two or more pathogenic variants in the *ABCA4* gene, where pathogenicity was determined by the genetic testing laboratory utilized for the given patient. In patients for whom segregation analysis was performed, variants were shown to be *in trans*. Patients with only one variant in *ABCA4* were excluded.

### Data collection

A review of electronic medical records was performed, and clinical data from baseline and from the most recent visit were collected for each patient. Clinical data included sex; estimated age of onset, based on when patients noticed first symptoms or when they were diagnosed; estimated disease duration, determined by the time between age of onset and the date when baseline images were acquired; visual acuity (VA) on the Snellen chart; ffERG responses; color fundus photography (CFP) imaging; fundus autofluorescence (FAF) imaging; and spectral domain optic coherence tomography (SD-OCT) imaging. The ffERG was performed according to the standards of the International Society for Clinical Electrophysiology of Vision (ISCEV). The ffERG results were divided into three groups, according to the classification system described by Mc Bain et al. [10]: group 1 consisted of patients with normal photopic and scotopic responses; group 2 consisted of subjects with reduced photopic responses only; and group 3 consisted of patients with reduced photopic and scotopic responses [10]. FAF was graded as one of three different types according to the classification system described by Fujinami et al. [11]: type 1 consisted of localized low signal at the fovea surrounded by a homogeneous background of autofluorescence; type 2 consisted of localized low signal at the macula surrounded by widespread foci of high and low signals extending anteriorly to the vascular arcades; and type 3 consisted of multiple areas of low signal throughout the posterior pole, extending beyond the vascular arcades. CFP images were analyzed for the presence of any sign of RPE clumping, defined as dark-black colored lesions within the posterior pole. SD-OCT images were analyzed for the presence of hyperreflective foci. The correlation of hyperpigmentary changes seen on CFP and the infra-red image from SD-OCT was overlaid with the SD-OCT image, using methodology similar to that described in a previous study [23]. Only hyperpigmented lesions with a corresponding SD-OCT B-scan were included. All data were obtained from the baseline visit. When available, data from the most recent follow up visits were also collected.

### Statistical analysis

Data were summarized into means and standard deviations for continuous variables and with counts and percentages for categorical variables. Student *t* test was used to compare visual acuity data at baseline and follow-up visits for those with macular hyperpigmentation only. Statistical analyses performed using SPSS, Version Pro 12. SAS Institute Inc., Cary, NC, 1989-2007.

## Results

### Patients characteristics

Of 141 patients with both a clinical and genetic diagnosis of STGD, 30 patients (21.27%) had macular hyperpigmentation observed on CFP. There were 15 men and 15 women, the mean age was 43.5 (standard deviation 16.4), the mean age of onset was 23 years (standard deviation 2.52), and the mean duration of disease was 19.9 years (standard deviation 15.1). Macular hyperpigmentation was observed in both eyes in 25 patients (83.3%). Baseline ffERG was available for 28 patients. The majority [17] of patients (60.7%) presented with reduction of photopic and scotopic responses (ffERG group 3), while 8 (28.5%) presented with reduction of photopic responses only (ffERG group 2), and 3 (5.1%) presented with preserved photopic and scotopic responses (ffERG group 1). FAF at baseline was available for 25 patients. Twelve patients (48%) presented with type 2, while eight (32%) presented with type 3, and 5 (20%) presented with type 1 autofluorescence.

### Characteristics of hyperpigmentary changes

**Fig. 1:**
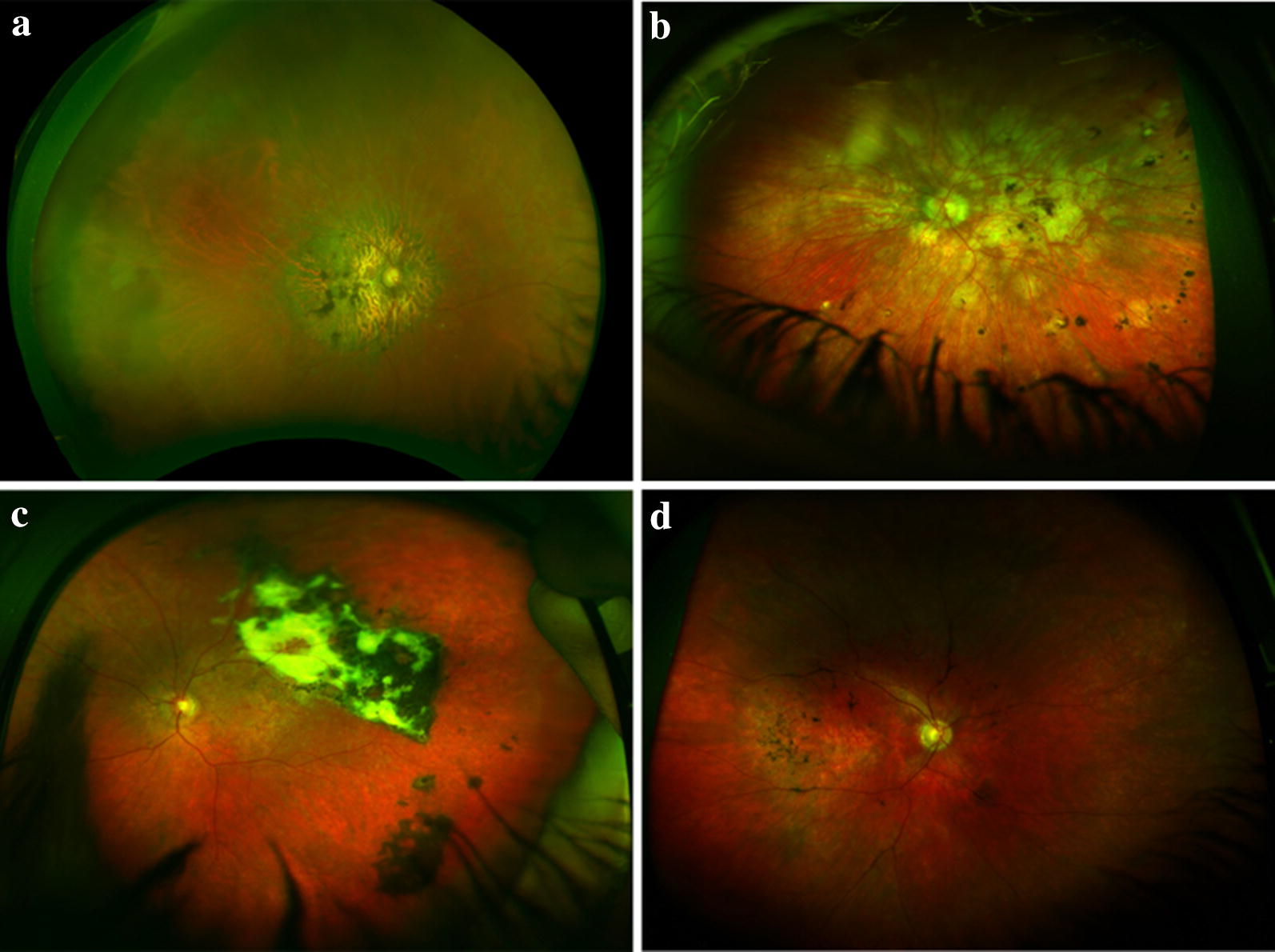
**a** Color photography of a patient with geographic atrophy and macular hyperpigmentation that is confined within areas of geographic atrophy (pattern 1). **b** Color photography of a patient with a diffuse hyperpigmentation in poorly-defined areas of atrophy (pattern 2). **c** Color photography of a patient with macular hyperpigmentation associated with subretinal fibrosis (pattern 3). **d** Color photography of a patient with perivenous pigment clumping throughout the macula (pattern 4) that also extends into the mid-periphery

Hyperpigmentation was observed in four distinct patterns in 55 eyes: pattern 1 consisted of focal areas of hyperpigmentation within well-demarcated areas of geographic atrophy; pattern 2 consisted of widespread RPE clumping within poorly demarcated atrophic areas; pattern 3 consisted of diffuse hyperpigmentation associated with subretinal fibrosis; and pattern 4 consisted of hyperpigmentation along retinal vasculature (perivenous pigmentation). In 35 eyes (63.6%), hyperpigmentation was associated with pattern 1, while in 18 eyes (51.4%) it was associated with pattern 2. Pattern 3 was observed in only 2 (3.6%) eyes. Perivenous hyperpigmentation was observed concomitantly in 10 eyes of patients with pattern 2. Representative images of the four patterns are shown in Fig. 1.

**Fig. 2:**
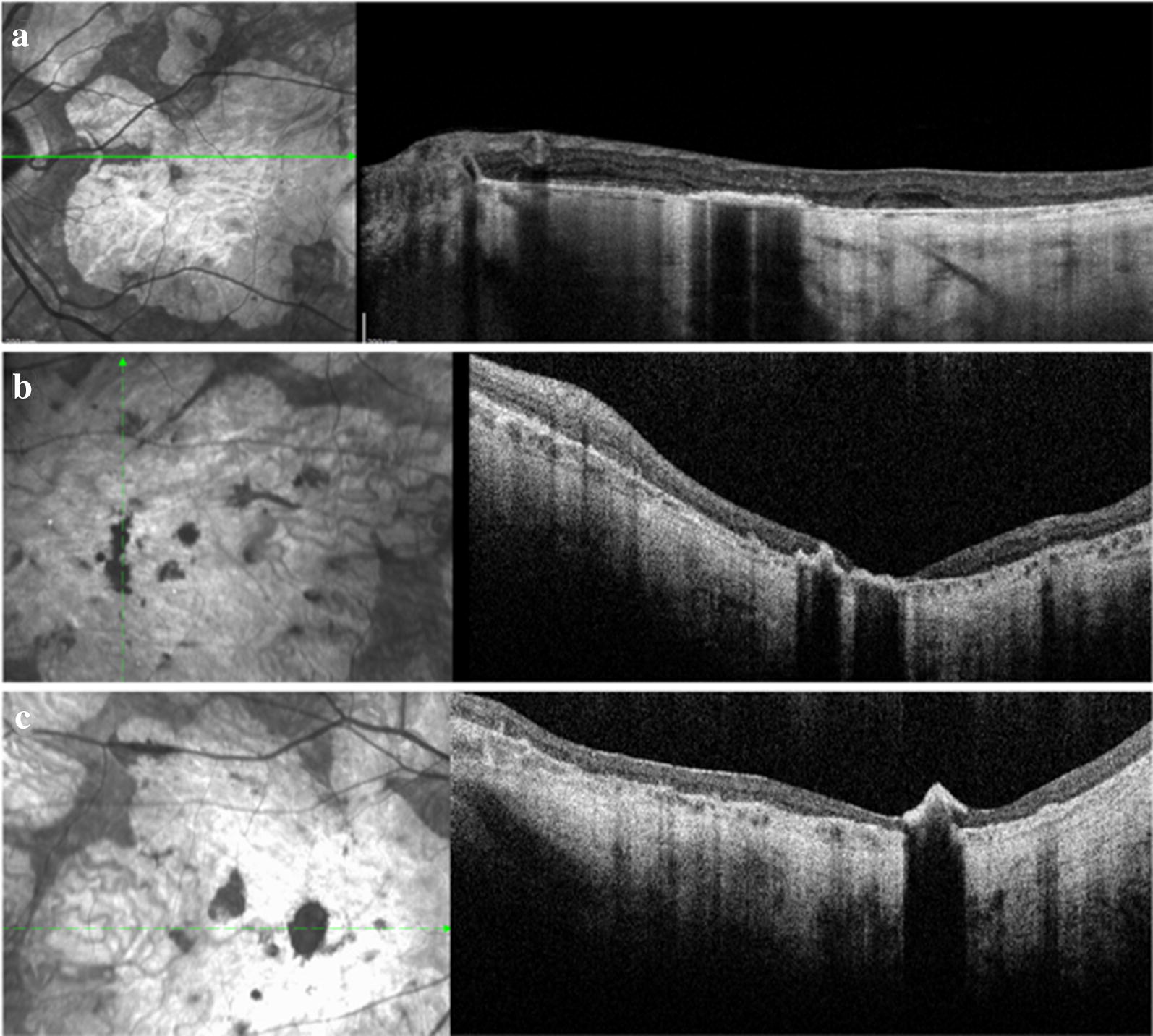
**a** SD-OCT shows hyperpigmented lesion localized in the outer retina. **b** SD-OCT shows hyperpigmented lesions located in the outer retina and pilling up towards the inner retina. **c** SD-OCT shows hyperpigmented lesion mostly located in the inner retina

SD-OCT was available for 22 patients (41 eyes). Eight patients and one eye were excluded from the SD-OCT analysis because the SD-OCT B-scans did not pass through the hyperpigmented lesions or because the hyperpigmented lesions seen on CFP were not clearly identified on infra-red image on SD-OCT. Hyperpigmentation appeared as hyperreflective and highly back-scattering lesions. In 31 eyes, hyperpigmentation was observed in the outer retina (comprising the IS/OS and RPE), while in 8 eyes, it was observed in the INL, ONL, ganglion cell layer (GCL) or retinal nerve fiber layer (RFNL) in addition to the IS/OS and RPE. Subretinal hyperpigmentation was observed in 2 eyes. SD-OCT images are shown in Fig. 2.

### Progression

Seventeen (56.6%) patients had CFP follow-up images. Eleven patients (64.7%) demonstrated increases (for both distribution and density) of the hyperpigmented lesions, along with progression of the underlying RPE atrophy, between the baseline visit and the most recent visit (mean follow up of 6.58 years). In 8 eyes, the pigment observed in the outer retina expanded to the inner layers, including the INL, ONL, GCL or RNFL on SD-OCT. VA remained stable. There was no statistically significant difference between the mean VA at the baseline (0.935 logMar) and the mean VA at the most recent visit (1.29 logMar) (*p* = 0.21).

## Discussion

In this study, it has been demonstrated that patients with STGD with hyperpigmented lesions in the posterior pole had a long disease duration and severe phenotype. This was confirmed by a poor VA, widespread disease on FAF, and photopic and scotopic dysfunction on ERG in those with MH. In addition, over time, hyperpigmented lesions increased in size, spread across the retina, and migrated to different retinal layers, as seen by SD-OCT. Macular hyperpigmentation may be a marker of advanced stage of the disease, when diffuse RPE atrophy leads to pathologic remodeling and clumping of the remaining RPE cells. Similarly, the development of peripheral pigmentation in patients with STGD has been previously associated with a severe phenotype and advanced disease stage. Previous studies have demonstrated the presence of peripheral pigmented retinal lesions resembling CHRPE lesions in a subset of patients with STGD disease who also had a more severe phenotype [24]. Therefore hyperpigmentation, whether in the macula or periphery, may be a marker of end-stage pathologic remodeling of the retina after many years of degeneration.

In patients with STGD, hyperpigmentation has been described following human embryonic stem cell-derived RPE (hESC-RPE) transplantation. In most eyes, hyperpigmentation was observed as subretinal hyperreflective foci at the border of atrophic areas, consistent with location of hyperpigmentation in the RPE-transplanted cells [25, 26]. In a few eyes that underwent hESC-RPE transplantation, a patch of epiretinal pigmentation also developed, which likely resulted from a reflux of the implanted material from the subretinal space or from injection into the pre-retinal space [25, 26]. In retinal dystrophies overall, RPE proliferation seems to occur in response to loss of surrounding RPE cells, which in turn leads to further retinal cell damage and loss [27, 28]. In our study, hyperpigmented lesions increased in size, spread across the retina, and migrated to different retinal layers over time. Therefore, it is possible that, similar to the pathogenesis of hyperpigmentation in patients with AMD and other retinal dystrophies, the hyperpigmentation observed in patients with STGD may also occur in response to loss of surrounding RPE cells, which in turn leads to further RPE damage and loss [27, 28], resulting in RPE degeneration, displacement and migration [19–21].

In AMD, macular hyperpigmentation occurs after extensive loss of the RPE and increases the risk of progression to either geographic atrophy or choroidal neovascularization [22]. Comparisons have been made between patients with AMD and STGD in part due to the similar pathogenesis that characterizes these degenerative diseases [4–6]. Interestingly, the presence of a single heterozygous mutation in *ABCA4* has been associated with an increased risk for AMD [29]. Considering that the use of RPE stem cell therapies are being investigated for both of these diseases, and that pathologic RPE remodeling is an important disease mechanism in both diseases, a robust understanding of RPE remodeling is necessary to anticipate possible outcomes for patients with STGD who undergo a therapeutic intervention. In STGD, macular hyperpigmentation may not be predictive or causative of a severe phenotype, but it may be a finding associated with an advanced stage of disease, after extensive photoreceptor degeneration and RPE atrophy have occurred. The relationship between RPE atrophy, photoreceptor degeneration, and RPE remodeling in both of these macular diseases may elucidate more about the etiology of macular hyperpigmentation and lead to potential treatments that might reverse the pathologic remodeling seen in these degenerative retinal diseases.

The limitations of this study include its retrospective design and limited number of visual functional tests. The absence of change in VA between baseline and follow up visits may have occurred because patients with STGD are known to have oscillation of the preferred retinal locus and have also been shown to reach a relative plateau in visual acuity at 20/200 [30–33]. In addition, in many cases, the areas of hyperpigmentation were outside the fovea, and, therefore, the effect of the lesions on visual function could not be evaluated only by measuring the central VA. The areas of geographic atrophy were also subjectively quantified, and, therefore, the association between progression of geographic atrophy and development of hyperpigmentary changes cannot be established.

Since cell and gene therapies are being developed for inherited retinal dystrophies, we need to be able to stratify patients by severity and progression of disease in order to optimize the ability to elicit successful responses to therapy. Clinical trials evaluating efficacy of novel therapies for patients with STGD may wish to document the presence of hyperpigmentation at baseline as well as after therapy. Furthermore, studies assessing other functional testing, such as microperimetry and contrast sensitivity, may provide insight into how these hyperpigmentary changes are associated with other visual function measures.
